# Methodological Insights on Recruitment and Retention From a Remote Randomized Controlled Trial Examining the Effectiveness of an Alcohol Reduction App: Descriptive Analysis Study

**DOI:** 10.2196/51839

**Published:** 2024-01-05

**Authors:** Melissa Oldham, Larisa Dinu, Gemma Loebenberg, Matt Field, Matthew Hickman, Susan Michie, Jamie Brown, Claire Garnett

**Affiliations:** 1 University College London London London United Kingdom; 2 Department of Psychology University of Sheffield Sheffield United Kingdom; 3 Population Health Sciences Bristol Medical School University of Bristol Bristol United Kingdom; 4 Centre for Behaviour Change University College London London United Kingdom

**Keywords:** alcohol reduction, alcohol, digital care, digital intervention, ethnic minority, methods, mHealth, randomised controlled trial, recruitment, retention, social media

## Abstract

**Background:**

Randomized controlled trials (RCTs) with no in-person contact (ie, remote) between researchers and participants offer savings in terms of cost and time but present unique challenges.

**Objective:**

The goal of this study is to examine the differences between different forms of remote recruitment (eg, National Health Service [NHS] website, social media, and radio advertising) in the proportion of participants recruited, demographic diversity, follow-up rates, and cost. We also examine the cost per participant of sequential methods of follow-up (emails, phone calls, postal surveys, and postcards). Finally, our experience with broader issues around study advertising and participant deception is discussed.

**Methods:**

We conducted a descriptive analysis of 5602 increasing-and-higher-risk drinkers (Alcohol Use Disorders Identification Test score ≥8), taking part in a 2-arm, parallel group, remote RCT with a 1:1 allocation, comparing the intervention (Drink Less app) with usual digital care (NHS alcohol advice web page). Participants were recruited between July 2020 and March 2022 and compensated with gift vouchers of up to £36 (a currency exchange rate of £1=US $1.26988 is applicable) for completing follow-up surveys, with 4 stages of follow-up: email reminders, phone calls, postal survey, and postcard.

**Results:**

The three main recruitment methods were advertisements on (1) social media (2483/5602, 44.32%), (2) the NHS website (1961/5602, 35.01%), and (3) radio and newspapers (745/5602, 13.3%), with the remaining methods of recruitment accounting 7.37% (413/5602) of the sample. The overall recruitment cost per participant varied from £0 to £11.01. Costs were greater when recruiting participants who were men (£0-£28.85), from an ethnic minority group (£0-£303.81), and more disadvantaged (£0-£49.12). Targeted approaches were useful for recruiting more men but less useful in achieving diversity in ethnicity and socioeconomic status. Follow-up at 6 months was 79.58% (4458/5602). Of those who responded, 92.4% (4119/4458) responded by email. Each additional stage of follow-up resulted in an additional 2-3 percentage points of the overall sample being followed up, although phone calls, postal surveys, and postcards were more resource intensive than email reminders.

**Conclusions:**

For remote RCTs, researchers could benefit from using a range of recruitment methods and cost-targeted approaches to achieve demographic diversity. Automated emails with substantial financial incentives for prompt completion can achieve good follow-up rates, and sequential, offline follow-up options, such as phone calls and postal surveys, can further increase follow-up rates but are comparatively expensive. We also make broader recommendations focused on striking the right balance when designing remote RCTs. Careful planning, ongoing maintenance, and dynamic decision-making are required throughout a trial to balance the competing demands of participation among those eligible, deceptive participation among those who are not eligible, and ensuring no postrandomization bias is introduced by data-checking protocols.

## Introduction

Randomized controlled trials (RCTs) are used to examine the efficacy of interventions on a wide range of health-related behaviors and outcomes [1-4]. RCTs examining the efficacy of digital interventions are increasingly taking place on the web or remotely. Web-based trials feature no in-person contact between researchers and participants, with the administration of the intervention and all measures completed on the web. Remote trials also have no in-person contact between researcher and participant but may involve some offline follow-up options, such as completing surveys over the phone or by post. Web-based and remote trials can be cheaper and less labor-intensive than in-person trials, although they present some unique challenges around recruitment, retention, and participant deception. Here, we present methodological insights from a large-scale (n=5602) remote RCT examining the effectiveness of a digital intervention, the “Drink Less” app [5], in helping increasing-and-higher-risk drinkers (Alcohol Use Disorders Identification Test [AUDIT] score ≥8) to reduce their alcohol consumption.

Digital interventions, such as websites and apps, are increasingly being used for a wide range of health behaviors [6] and can offer benefits over in-person interventions in terms of cost, convenience, and anonymity [7]. RCTs aiming to evaluate digital interventions can be conducted on the web or remotely and may have several advantages relative to trials requiring in-person contact. First, web-based and remote settings could increase the external validity of the trial, as having to travel to in-person appointments for baseline or follow-up assessments does not reflect real-world implementation or how users access digital interventions [8]. Second, in theory, participants can be recruited from throughout nations or even globally, giving a larger and potentially more generalizable sampling frame [9]. Third, the cost of web-based or remote trials is likely to be much less as they can be partly automated, reducing demands on researcher time, and could potentially reduce researcher bias through double blinding [9].

However, there are also significant challenges with web-based or remote RCTs beyond those conducted in person. First, it may be harder to recruit participants or to recruit a broadly representative sample [10,11], as some groups, such as older adults and people from less advantaged communities, may be less likely to engage with research conducted remotely [12]. Second, researchers have less control over who signs up, and it is possible that motivated individuals may sign up multiple times for financial incentives [8,13]. Third, once recruited, researchers may have less control over how participants engage with the intervention [14] or respond to follow-ups [8]. This could be particularly problematic with groups who may have low digital literacy and may not understand how to use the intervention, although this may be reflective of how people would engage with digital interventions in real-world settings. There are other challenges that are present in both remote and in-person trials. Contamination occurs when the comparator group finds the intervention being tested outside of the trial [9]. This could be particularly likely if the comparator group receives an intervention they do not deem acceptable and seeks out alternatives. These biases could introduce bias into RCTs, which could obscure the effect of the intervention.

Here, we draw on data from a large-scale remote RCT, evaluating the effectiveness of the Drink Less app [5] compared with usual digital care (the National Health Service [NHS] alcohol advice web page). Drink Less is a theory- and evidence-informed, app-based intervention designed by researchers [15,16] to help increasing-and-higher-risk drinkers reduce their alcohol consumption. To mitigate some of the potential challenges outlined above, the trial used a multipronged recruitment strategy, including an advertisement on the NHS website and social media advertising [5]. In line with previous research [11], and to maximize follow-up rates, we offered substantial financial incentives to complete follow-up surveys, including an additional amount for completing the primary outcome within the first 24 hours, and undertook a comprehensive follow-up approach by sequentially sending follow-up reminders through email, SMS text messages, and telephone and by post. These strategies and broader methodological issues will be discussed ahead.

This study aims to:

1. Compare different remote recruitment methods in terms of cost per recruited participant, retention rates, participant deception, and sociodemographic diversity.
2. Compare the proportion of returned responses using different strategies for follow-up at each time point, and compare the cost and time associated with each follow-up stage.
3. Consider broader methodological issues pertaining to recruitment, retention, and participant deception, and discuss the success of strategies to mitigate these issues throughout the trial.

## Methods

The protocol and analysis plan were preregistered on the Open Science Framework [17]. The trial was registered (ISRCTN64052601). The main trial findings are reported elsewhere [18].

### Design

Descriptive secondary data analysis of a remote RCT [5] evaluating the effectiveness of the digital intervention “Drink Less” in reducing alcohol consumption in increasing-and-higher-risk drinkers.

### Participants

A total of 5602 participants were randomized in the RCT evaluating Drink Less. Participants were eligible if they were aged 18 years or older, lived in the United Kingdom, were increasing-and-higher-risk drinkers (AUDIT score ≥8) [19], had access to an iOS device (iPhone, iPod touch, or iPad), and wanted to drink less alcohol. Recruitment ran from July 2020 to March 2022 and included an advertisement on the NHS website, a mail-out to a database of UK-based users of the smoking cessation app “Smoke Free”, radio and social media advertising, press releases, and local advertising through health care providers. Advertisements were codeveloped with public representatives.

Informed consent was sought at baseline to participate in 3 web-based follow-up surveys at 1, 3, and 6 months. Surveys were completed on the web through Qualtrics (Silver Lake), although at the 6-month follow-up, offline options (eg, phone and post) were available. The 6-month follow-up survey assessed primary and secondary outcomes relating to alcohol use and a range of related measures. The 1- and 3-month follow-up surveys only assessed secondary outcome measures relating to alcohol use. We attempted to contact participants within 30 days of their first invitation to complete each follow-up survey. To maximize data retention and to allow for time taken for answers to be posted at 6-month follow-up, data provided up to 2 weeks after the 30-day period were accepted.

Initially, as well as through 3 emails (days 0, 5, and 9) and (from January 15, 2022) a total of 2 SMS text messages (days 5 and 9), we had planned that at the 1-, 3-, and 6-month follow-up, all participants would also be sequentially offered opportunities to complete follow-up through phone (called twice from days 10 to 17), a mailed survey (from day 18), and a mailed postcard (from day 30). However, due to resource constraints, from November 2020 on, we only used automated emails on days 0, 5, 9, and 11 to contact participants at the 1- and 3-month follow-up; we no longer called or sent postal surveys. With the aim of improving these follow-up rates with less resource, we added SMS text messaging follow-ups. Phone calls, mailed surveys, and postcard follow-ups were retained for the 6-month follow-up survey (when the primary outcome was measured).

### Measures

#### Recruitment Method

At baseline, participants were asked to specify where they saw the study advertised, with the following response options: NHS website, social media (eg, Facebook and Twitter [subsequently rebranded X]), other media (eg, radio and newspapers), emailed by the Smoke Free app, local health care provider, word of mouth, Google, general practitioner (GP) surgery, or other. If they selected “other,” free-text responses that fell within one of the response options were recoded (eg, Facebook would be social media). The response options “local health care provider” and “GP surgery” were collapsed. Throughout the study, both untargeted and targeted (eg, at men) social media advertisements were used. These were analyzed separately.

#### Participant Deception

We experienced 3 distinct subgroups of participant deception throughout the trial: duplicates, manual fraud, and bots. Duplicate responses, where individuals signed up more than once with identical names and phone numbers, were the least prevalent (n=49) and easiest to detect. Data checks were undertaken each month to search for duplicate values. Manual fraud was a more prevalent form of participant deception (n=297), defined as individuals who signed up multiple times with false information, such as phone numbers linked to businesses where they were not known or addresses that did not exist. To identify manual fraud, monthly checks were made on all addresses and telephone numbers provided to ensure street names matched the postcode and that numbers were mobile phone numbers. Any suspicious responses were flagged, and the participants were contacted and asked to confirm their details over the phone. Where individuals were not known at the phone number provided, they were removed from the study. To make it easier to automatically screen out those engaging in manual fraud, we added attention checks, whereby individuals were asked to select a certain response option. Participants were also asked to confirm their age at 2 different points in the baseline survey to ensure they were consistent. Individuals failing either of these attention checks were screened out of the survey before randomization. The most prevalent type of fraud were “bot” responses (n=863). These were fraudulent responses similar to manual fraud, but they occurred in batches of 20-30 at a time when contact information was given in noticeably similar formats (eg, firstname123@emailaddress.com), often with American street addresses (being UK-based was an inclusion criteria of the trial). These responses seemed to be automated and were identified using the same process of address checking as above (individuals not known at the phone number provided were removed from the study). Adding a CAPTCHA (Completely Automated Public Turing test to tell Computers and Humans Apart) to the survey eliminated this issue. A more detailed discussion on participant deception is described elsewhere [20].

#### Sociodemographic Characteristics

Sociodemographic measures were assessed at baseline. This study focuses on gender, ethnicity, and occupation (to derive socioeconomic status [SES]: ABC1 [managerial, professional, and intermediate occupations] versus C2DE [skilled, semiskilled, unskilled manual, and lowest-grade worked or unemployed]).

### Analysis

#### Aim 1: Methods of Recruitment

Each recruitment method is compared in terms of the proportion of enrolled participants, the proportion of participants who were men, from a minority ethnic group, or from a more disadvantaged background (C2DE), and the proportion of participant deception. Cost-per-recruited participant citing each recruitment method (eg, total spend on recruitment method divided by the number of participants citing recruitment method) is reported. As well as the overall cost per participant, we also present the cost per participant stratified by gender (eg, for each man recruited), ethnicity (eg, for those from ethnic minority individuals), and SES (eg, for those from more disadvantaged backgrounds). Finally, we present follow-up rates at 1-, 3-, and 6-months for each method of recruitment.

#### Aim 2: Follow-Up

The proportion of the sample responding at each sequential stage of follow-up (ie, emails, phone calls, postal surveys, and postcards) is reported. The cost of each follow-up stage per participant responding at each stage is also reported. This was derived by dividing the estimated researcher time and other relevant costs by the number of follow-ups completed at each stage.

#### Aim 3: Broader Methodological Issues

Broader methodological issues such as advertising, participant deception, technical support, contamination, and boosting retention are discussed. We describe and briefly discuss the strategies we used throughout the trial to mitigate issues.

### Ethical Considerations

Ethical approval for the iDEAS (iOS Drink Less, Evaluating the Effectiveness of an Alcohol Smartphone app) trial was granted by the University College London (UCL) Ethics Committee (16799/001). Participants provided informed consent before participating in the trial. Study data were pseudoanonymized and stored on a secure university drive. Participants were compensated with gift vouchers of up to £36 (a currency exchange rate of £1=US $1.26988 is applicable) for completing the 3 surveys: £6 for the survey at 1 and 3 months and £12 at 6 months, with an additional £12 if the 6-month survey was completed within 24 hours.

## Results

### Sample Characteristics

A total of 5602 participants completed the baseline survey between July 2020 and March 2022: 65.78% (3685/5602) responded at 1-month follow-up, 63.80% (3574/5602) at 3-month follow-up, and 79.58% (4458/5602) at 6-month follow-up. Over half (3207/5602, 57.25%) of the sample were women, 42.22% (2365/5602) were men, 0.46% (26/5602) were “other,” and 0.07% (4/5602) preferred not to say. Most of the sample were White (5296/5602, 94.54%) and earned above-average income (4151/5602, 74.01%). The sample characteristics were similar at each follow-up. Table 1 reports the sociodemographic characteristics of the sample at baseline and those responding at each stage of follow-up.

**Table 1:** Sample characteristics at baseline and among those who responded at 1-month, 3-month, and 6-month follow-up for increasing-and-higher-risk drinkers participating in the iDEAS (iOS Drink Less, Evaluating the Effectiveness of an Alcohol Smartphone) randomized controlled trial (RCT).

Variable		Baseline^a^ (N=5602)		1-month follow-up (n=3685)		3-month follow-up (n=3574)		6-month follow-up^a^ (n=4458)
**Gender, n (%)**								
	Men	3207 (57.25)	2046 (55.52)		1992 (55.74)		2534 (56.84)	
	Women	2365 (42.22)	1620 (43.96)		1565 (43.79)		1903 (42.69)	
	Other	26 (0.46)	16 (0.43)		14 (0.39)		17 (0.38)	
	Prefer not to say	4 (0.07)	3 (0.08)		3 (0.08)		4 (0.09)	
**Ethnicity, n (%)**								
	Asian	96 (1.71)	68 (1.85)		68 (1.9)		83 (1.86)	
	Black	47 (0.84)	35 (0.95)		39 (1.09)		41 (0.92)	
	Chinese	9 (0.16)	9 (0.24)		9 (0.25)		9 (0.2)	
	White	5296 (94.54)	3474 (94.27)		3361 (94.04)		4206 (94.35)	
	Mixed	113 (2.02)	75 (2.03)		71 (1.99)		84 (1.88)	
	Other	21 (0.37)	15 (0.41)		15 (0.42)		18 (0.4)	
	Prefer not to say	19 (0.34)	9 (0.24)		11 (0.31)		16 (0.36)	
	Not known	1 (0.02)	0 (0)		0 (0)		1 (0.02)	
**Occupation, n (%)**								
	ABC1^b^	4151 (74.01)	2759 (74.87)		2688 (75.21)		3337 (74.85)	
	C2DE^c^	1451 (25.9)	926 (25.13)		886 (24.79)		1121 (25.15)	

^a^The data is also reported in the main trial paper [18].

^b^ABC1: managerial, professional, and intermediate occupations.

^c^C2DE: skilled, semiskilled, unskilled manual, and lowest-grade worked or unemployed.

### Aim 1: Recruitment Methods, Demographic Diversity, and Cost Per Participant

Most participants recruited for this trial reported seeing it advertised on social media (2483/5602, 44.32%), the NHS website (1961/5602, 35.01%), or through radio or newspapers (745/5602, 13.3%), with all other recruitment methods accounting for 7.37% (413/5602) of the sample (Table 2).

**Table 2:** Total recruitment and proportion of recruited sample of iDEAS (iOS Drink Less, Evaluating the Effectiveness of an Alcohol Smartphone) randomized controlled trial who were men, of minority ethnic groups, had lower socioeconomic status (SES), and identified as a fraudulent response by recruitment method.

Recruitment method	Included sample (N=5602), n (%)	Men, n (%)^a^	Ethnic minority group, n (%)^a^	Low SES, n (%)^a^	Fraudulent response, n/N (%)^b^
Untargeted social media	2119 (37.82)	650 (30.67)	147 (6.94)	507 (23.93)	1020/3139 (32.49)
Targeted social media	364 (6.5)	353 (96.98)	13 (3.57)	90 (24.73)	8/372 (2.15)
National Health Service website	1961 (35.01)	628 (32.02)	76 (3.88)	570 (29.07)	123/2084 (5.9)
Radio or newspapers	745 (13.3)	591 (79.33)	27 (3.62)	167 (22.42)	19/764 (2.49)
Word of mouth	142 (2.53)	74 (52.11)	9 (6.34)	41 (28.87)	11/153 (7.19)
Google	159 (2.84)	44 (27.67)	7 (4.4)	50 (31.45)	9/168 (5.36)
Smoke Free email	55 (0.98)	13 (23.64)	3 (5.45)	11 (20)	10/65 (15.38)
Health care provider or general practitioner	15 (0.27)	5 (33.33)	3 (20)	4 (26.67)	16/31 (51.61)
Other	42 (0.75)	7 (16.67)	2 (4.76)	11 (26.19)	0/42 (0)

^a^The percentage of participants recruited from each method (ie, N=*Included sample* value).

^b^The percentage of the overall sample from each recruitment method including those removed after participant deception checks.

Ongoing sociodemographic tracking throughout the study revealed that women, White, and advantaged participants were being overrecruited. In response, strategies targeted at a more diverse sample in terms of gender, SES, and ethnicity were introduced with mixed success. This included targeted social media advertisements aimed at men and radio advertisements on Talk Radio, Asian Sounds (in English and Urdu), and Punjabi Radio (in English and Punjabi).

Recruitment methods differed in the proportion of men (range 17%-97%), with targeted approaches including social media advertising (353/364, 97% men) and radio advertising (591/745, 79.3%) being the most successful in recruiting a sample of men. Word of mouth was most effective in terms of recruiting a balanced sample in terms of gender (74/142, 52.1% men) but recruited a small proportion (142/5602, 2.53%) of the sample overall.

Recruiting through GP surgeries and local health care providers resulted in the highest proportion of participants from minority ethnic groups (3/15, 20%) but recruited a small proportion of participants in total (15/5602, 0.27%). Untargeted social media advertisements and word of mouth were the next best, with 6.94% (147/2119) and 6.3% (9/142) of the sample coming from ethnic minority individuals, respectively.

The NHS website, word of mouth, and Google all recruited around a third of participants who were more disadvantaged. However, both Google (159/5602, 2.84%) and word of mouth (142/5602, 2.53%) recruited a small proportion of participants in total.

The final column of Table 2 refers to the proportion (and number) of participants who were removed from the study due to participant deception, citing each recruitment method. A total of 84.54% (1028/1216) of participants identified as fraudulent cited social media as the place they saw the advertisement. It should be noted here that these participants may not have been honest in terms of where they saw the study advertisement and may have been deliberately misreporting where they found the study or responding at random

Money spent on each of the recruitment methods varied from £0 for the NHS advertisement and word of mouth to £8203 for radio or newspaper advertisements (Table 3; a currency exchange rate of £1=US $1.26988 is applicable). Of the paid forms of recruitment, social media advertising and advertising through health care providers were the cheapest ways of recruiting participants who were men, of ethnic minorities, or from more disadvantaged backgrounds.

Although the overall number of participants recruited from health care settings was low, this was impeded by the COVID-19 pandemic. The initial recruitment plan was to have posters in primary care surgeries throughout the United Kingdom; however, due to the pandemic and associated lockdowns for most of the recruitment period, many people received health care on the web and were not visiting GP surgeries. We only started advertising in GP surgeries for the last 5 months of trial recruitment (in November 2021).

Those recruited from health care providers (15/15, 100%), Smoke Free email (51/55, 93%), and word of mouth (126/142, 88.7%) appeared to have the highest response rates and those recruited through advertisements on Google (109/159, 69%), and the NHS website (1513/1961, 77%) appeared among the lowest. Table 4 presents the follow-up rates at 1-, 3-, and 6-month follow-up.

**Table 3:** Total cost per participant and cost per participant who were men, of ethnic minority groups, and lower socioeconomic status (SES) by recruitment method for participants of the iDEAS (iOS Drink Less, Evaluating the Effectiveness of an Alcohol Smartphone) randomized controlled trial. A currency exchange rate of £1=US $1.26988 is applicable.

Recruitment method	Recruited, n	Total cost (£)	Cost per participant (£)	Cost per man (£)	Cost per ethnic minority participant (£)	Cost per low SES participant (£)
Untargeted social media	2119	6750.00	3.19	10.38	45.92	13.31
Targeted social media	364	690.00	1.90	1.95	53.08	7.67
National Health Service website	1961	0	0	0	0	0
Radio or newspapers	745	8203.00	11.01	13.88	303.81	49.12
Word of mouth	142	0	0	0	0	0
Google	159	1247.00	7.84	28.34	178.14	138.56
Smoke Free email	55	375.00	6.82	28.85	125.00	34.09
Health care provider or general practitioner	15	61.00	4.07	12.20	20.33	15.24
Other	42	0	0	0	0	0

**Table 4:** Follow-up rates at 1-, 3-, and 6-month follow-up among increasing-and-higher-risk drinkers participating in the iDEAS (iOS Drink Less, Evaluating the Effectiveness of an Alcohol Smartphone) randomized controlled trial by recruitment method.

Recruitment method	Recruited, n	Follow-up rate at 1 month, n (%)	Follow-up rate at 3 months, n (%)	Follow-up rate at 6 months, n (%)
Untargeted social media	2119	1376 (64.94)	1340 (63.24)	1708 (80.6)
Targeted social media	364	277 (76.1)	256 (70.33)	295 (81.04)
National Health Service website	1961	1237 (63.08)	1210 (61.7)	1513 (77.15)
Radio or newspapers	745	520 (69.8)	495 (66.44)	603 (80.94)
Word of mouth	142	107 (75.35)	106 (74.65)	126 (88.73)
Google	159	87 (54.72)	81 (50.94)	109 (68.55)
Smoke Free email	55	38 (69.09)	43 (78.18)	51 (92.73)
Health care provider or general practitioner	15	12 (80)	10 (66.66)	15 (100)
Other	42	31 (73.81)	33 (78.57)	38 (90.48)

### Aim 2: Retention During Sequential Follow-Up

At 6-month follow-up, 92.4% (4119/4458) of those who responded did so in response to 1 of the 3 email notifications. An additional 2.02% (90/4458) responded following 2 phone calls from the research team, and 3.25% (145/4458) responded following a postal survey. The final stage of recruitment, a postcard sent through mail to participants featuring just the key outcome measure for the trial (AUDIT-C), yielded a further 2.33% (104/4458) of the followed-up sample. The estimated costs of each sequential stage of follow-up are presented in Tables 5 and 6.

**Table 5:** A summary of time spent and cost on different stages of contacting participants of the iDEAS (iOS Drink Less, Evaluating the Effectiveness of an Alcohol Smartphone) randomized controlled trials at 1- and 3-month follow-up. A currency exchange rate of £1=US $1.26988 is applicable.

Time point and method		Total follow-up sent, n	Follow-up per month, mean (range)	Responded	Hours spent sending follow-up^a^	Hours spent sending vouchers	Total research hours	Cost research hours^b^ (£)	Other costs (£)	Total cost (£)	Cost per participant (£)
**1-month follow-up**											
	Automated email	5602	267 (65-621)	1874	0	34	34	672	0	672	0.36
	First manual email and SMS text message^c^	3800 and 1057	181 (34-448)	1130	130	20	150	2966	106^d^	3072	2.72
	Second and third manual email and SMS text message^c^	3291 and 648	175 (0-462)	643	112	12	124	2452	65^d^	2517	3.91
**3-month follow-up**											
	Automated email	5602	267 (65-621)	2053	0	37	37	732	0	732	0.36
	First manual email and SMS text message^c^	3610 and 1282	172 (26-419)	1056	123	19	142	2807	128^d^	2935	2.78
	Second and third manual email and SMS text message^c^	3698 and 874	176 (0-511)	460	126	8	134	2649	87^d^	2736	5.95

^a^Time spent sending manual reminders and SMS text messages. On average, an email and SMS text message reminder took 2 minutes and 5 seconds to send, and a voucher email took 1 minute and 8 seconds to send.

^b^The cost here is the average of 2 research staff salaries (£19.77) × research hours.

^c^For the first 3 months of follow-up, we contacted participants twice manually by email, followed sequentially by phone calls, a written survey, and a postcard with the primary outcomes. However, this was not sustainable, so the subsequent follow-up stages were dropped at 1 and 3 months and are not presented below but are included in this total. 1-month phone completions=22, and 1-month postcard completions=16. 3-month phone completions=4, and 3-month postcard completions=1. SMS text messages were added 18 months into recruitment and sent at the same time as the first and second emails, so individual effects cannot be differentiated. SMS text messages did not add significantly to the time spent sending them, as they were also sent through mail merge at the same time.

^d^Based on 10 pence (US $0.12) per SMS text message.

**Table 6:** A summary of time spent and cost on different stages of contacting participants of the iDEAS (iOS Drink Less, Evaluating the Effectiveness of an Alcohol Smartphone) randomized controlled trial (RCT) at 6-month follow-up. A currency exchange rate of £1=US $1.26988 is applicable.

Method	Total follow-up sent, n	Follow-up per month, mean (range)	Responded (n=4458)	Follow-Up hours^a^	Voucher hours^b^	Data entry hours^c^	Total hours	Cost hours^d^ (£)	Other costs (£)	Total cost (£)	Cost per participant (£)
Email through Qualtrics	5602	266 (64-621)	2358	0	42	—^e^	42	830	0	830	0.35
First manual follow-up email and SMS text message^f^	1886 and 505	52 (4-132)	948	64	17	—	81	1601	51^g^	1652	1.74
Second manual follow-up email and SMS text message^f^	1077 and 450	51 (4-132)	813	37	15	—	52	1028	45	1073	1.32
Phone calls	2118	101 (8-260)	90	117	2	—	119	2353	0	2353	26.14
Posted survey	1378	66 (2-167)	145	68	3	24	95	1878	2384^h^	4262	29.39
Postcard	1161	55 (2-156)	104	59	2	9	70	1384	1080^i^	2464	23.69

^a^Based on average times of 2.05 minutes per email or SMS text message, 3.31 minutes per phone call, 2.94 minutes per survey, and 3.07 minutes per postcard.

^b^A voucher email took 1.08 seconds to send.

^c^Based on 10 minutes to input a survey and 5 minutes to input a postcard.

^d^The cost here is the average of 2 research staff salaries (£19.77) × research hours.

^e^Not available.

^f^Text messages were added 18 months (from January 15, 2022) into recruitment and sent at the same time as the first and second emails, so individual effects cannot be differentiated.

^g^Based on 10 pence (US $0.12) per SMS text message.

^h^Based on estimated stationary and postage costs of £1.73 per survey.

^i^Based on estimated stationary and postage costs of £0.93 per postcard.

### Aim 3: Broader Methodological Insights

#### Retention

Each SMS text message cost 10 pence (US $0.12) to send and required minimal researcher time as texts were sent to participants through mail merge at the same time as email reminders were sent. This was relatively low cost and low effort, and there was an increase in the average follow-up rate at 1- and 3-month follow-up in the 3 months before and after the introduction of the SMS text messages (from 58.0% (221/381) to 71.43% (830/1162) at 1 month and 58.5% (223/381) to 64.80% (753/1162) at 3 months).

#### Recruitment

Remote trials may unintentionally exclude participants with less experience using web-based surveys and digital interventions or with lower digital literacy. To mitigate this risk, in the recommendation email and at the end of the baseline survey, we included a link to a pictorial step-by-step guide to downloading and using the app [21] and encouraged participants to contact the research team if they needed technical support. Less than 10 participants contacted the research team for technical support throughout the trial.

#### Advertisement Development

Advertising any research study involves balancing incentivizing the target audience to participate while avoiding incentivizing those outside of the target market to falsify information to gain reimbursement. This is particularly true of remote research, where there is no face-to-face contact with researchers and therefore fewer barriers to participant deception. Below, we outline the process of developing the study advertisement, involving feedback from public and patient involvement (PPI) groups and dynamic changes throughout the trial in response to higher rates of participant deception.

##### PPI Feedback on Advertising

**Figure 1:**
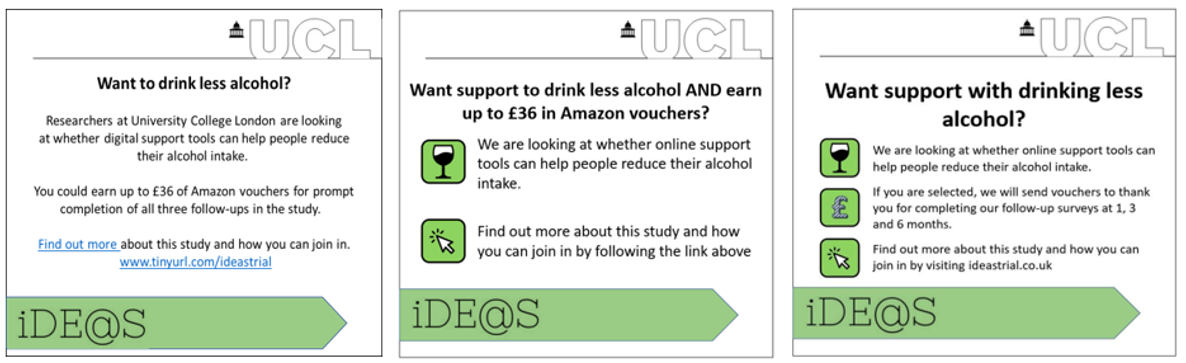
The original study advertisement designed by the research team to recruit participants to the iDEAS randomized controlled trial (left), advertisement following public and patient involvement feedback (middle), and advertisement following issues with participant deception (right). iDEAS: iOS Drink Less, Evaluating the Effectiveness of an Alcohol Smartphone; UCL: University College London.

To improve the clarity and appeal of the advertisement, we attended meetings with 2 PPI groups (the Sheffield Addiction Recovery Research Panel and the Alcohol and Food Discussion Group at the University of Stirling) and asked for feedback on an advertisement we had designed (Figure 1). The PPI group highlighted language (eg, “Researchers at UCL” and “trial”) that they felt was too formal and would make the study sound frightening or labor-intensive. Furthermore, they did not like the phrase “digital support tools,” which they felt was unclear, and instead suggested we use the phrase “online support tools.” The group also suggested that to make the advertisement more appealing, we should make it clear that people would get support to drink less alcohol, highlight the financial incentives in a more prominent position, and include pictures.

##### Advertising and Participant Deception

Following issues with participant deception, edits were made to the advertisement to disincentivize those who did not meet the inclusion criteria from signing up for financial reimbursement. The mention of the vouchers was removed from the heading and moved to the body of the advertisement. The specific amount was removed, and the text was updated to make it clear that there was no immediate financial incentive to participate in the study; rather, vouchers were sent after 1-, 3-, and 6-month follow-up surveys were completed.

##### Negative Engagement With Advertising

Throughout the study, we also experienced negative engagement with our social media advertising, particularly on Facebook. Unhelpful comments included those joking about wanting to drink more (eg, “I need support with drinking MORE alcohol”), leaving negative messages about the research team (eg, “killjoy weirdos”), highlighting the reimbursement amount (eg, “vouchers sound good”), or telling other users reasons they had been screened out (eg, “people who use android rather than apple ones are not wanted”). We decided against disabling comments on advertising posts, as other people used them to engage positively with the study and to tag friends. Rather than respond to or delete posts, which may have further antagonized people, we used the “hide” feature on negative comments on a weekly basis, meaning these comments could not be seen by others but that the original poster was not notified. A total of 46.6% (210/451) of comments were hidden throughout the study.

#### Contamination

This was a pragmatic trial, as we were testing the effect of the recommendation rather than the use of the Drink Less app. Nevertheless, we took steps to minimize contamination. We were careful not to mention the name of the app or the trial in any advertising. We also included 2 sensitivity analyses to try and capture the extent of contamination in the trial. One focused on those who followed the recommendation determined by self-report (at 1-month and 6-month follow-ups). The second was an instrumental variable analysis that accounted for nonuse in the intervention group and contamination in the comparator group by operationalizing the difference in app use between the 2 groups.

These recommendations are summarized in Textbox 1.

**Methodological recommendations for remote randomized controlled trials (RCTs).**

**Recruitment**

- Use a range of recruitment methods.
- Monitor the demographic composition of the sample during trials and have targeted methods for underrecruited groups.
- Targeted advertising on social media or radio can be successful in recruiting men and can yield large numbers of responses. Having advertisements run consecutively for weeks seemed to result in cumulative benefits.
- General practitioner (GP) surgeries and word of mouth were good for recruiting a more balanced sample in terms of gender, ethnicity, and socioeconomic status (SES) but overall yielded lower numbers of participants. However, these methods were likely impacted by the COVID-19 pandemic and may be more effective with an increased investment of time or money in future trials.
- Offer technical support for online surveys and intervention use, ideally in different forms such as through pictorial step-by-step guides or through phone or email to ensure recruitment and engagement are inclusive.

**Follow-up**

- Offline follow-up options, such as phone calls and postal surveys, are more resource intensive but can increase follow-up rates.
- SMS text messaging services can be a relatively low-cost and low-effort way of boosting follow-up rates.

**Advertising and incentives**

- Avoid overly formal language, which may alienate participants, and use pictures.
- Highlight benefits to participants other than financial incentives (eg, support for alcohol reduction).
- Tailor advertising strategies to ensure the right balance of incentivization across different platforms. For example, if advertising on social media or where barriers to sign up are low, mentioning incentives could result in motivated individuals falsifying information. However, where there are more barriers to sign up, for example, through a radio advertisement where participants must find the study link independently, it may be necessary to highlight incentives more explicitly.

**Participant deception**

- Be aware of different types of fraud and the best ways to detect them, and continuously monitor data as strategies are likely to evolve in response to checks and barriers introduced. These may include address checks, phone calls, or requiring participants to submit ID.
- When creating online surveys, researchers should use fraud detection software if it is offered (eg, CAPTCHAS [Completely Automated Public Turing test to tell Computers and Humans Apart]) and check licenses to see if additional fraud detection software is available.
- Include attention-check questions where participants are asked to give stable information at different points in a survey or where participants are asked to select a particular response option.
- Ensure costing is included for the data monitoring resources required.

**Contamination**

- Consider the inclusion of sensitivity analyses, such as instrumental variable analysis, to capture the extent of contamination in remote randomized controlled trials.

## Discussion

### Summary of Findings

In this remote RCT, the 3 main participant recruitment methods were through advertisements on social media (2483/5602, 44.32%), the NHS website (1961/5602, 35.01%), and through radio or newspapers (745/5602, 13.3%), with all other methods of recruitment accounting for 7.37% (413/5602) of the sample. More participants who were women, White, and from more advantaged backgrounds responded to the initial recruitment. Targeted approaches through social media and radio advertising were successful in recruiting men but less successful in appealing to a more diverse demographic in terms of ethnicity and SES. The most effective methods for recruiting more balanced samples (health care providers and word of mouth) were often responsible for a relatively small proportion of the overall sample, suggesting greater investment in these methods could be a positive strategy in future trials. The costs associated with different recruitment methods varied. There was an increase in cost per participant when recruiting participants who were men, from ethnic minorities, and from more disadvantaged backgrounds across all recruitment methods.

There was evidence that the sequential approach taken to 6-month follow-up was successful, with 79.58% (4458/5602) follow-up rates at 6-months. Most participants responded following automated emails and substantial financial incentives, including an additional incentive to respond to the primary outcome within the first 24 hours, but each additional stage of follow-up resulted in an additional 2% to 3% of the sample following up. The advantage of the sequential approach is also evidenced by the greater follow-up rate (4458/5602, 79.58%) at 6-month follow-up when this process was followed, relative to the follow-up rates at 1- and 3-months (3685/5602, 65.78% and 3574/5602, 63.8%, respectively), where only email or SMS text reminders were sent and less financial incentive was offered. However, each of the offline stages of follow-up was considerably more resource intensive than email reminders, so this is a practical consideration to be made at the costing stage. It would be of great interest to compare, across trials, the sociodemographic characteristics associated with the sample captured at each stage of follow-up. For example, it may be possible that offline stages of follow-up may be effective in retaining less digitally literate or less engaged participants.

### Implications

When making methodological decisions about remote RCTs, there is rarely a right answer that is applicable to every study or circumstance. It is important to be aware of balancing forces, which often pull in different directions. For example, when considering advertising, it is important to balance making the study appealing to the target market with not making the study so appealing that it yields a high rate of participants who sign up with false information or who respond multiple times to gain financial reimbursement. There is a similar trade-off when considering processes aimed at reducing participant deception in the data. It is important that processes that aim to ensure participants are real and eligible do not add postrandomization bias to remote RCTs by removing “real” participants in potentially nonrandom ways. Part of navigating this balance is to plan carefully and tailor decisions to individual circumstances, as well as to monitor and learn from decisions made throughout a trial.

### Previous Research

The findings of this study are in line with other studies that have focused on methodological issues in remote studies and RCTs [11,13]. The recruitment strategy undertaken was informed by a previous smoking cessation trial, which recommended using a range of sources but also monitoring the success of strategies throughout to recruit a large, diverse sample [11]. We have reported on the success of each strategy here to inform the planning of future trials. An additional potential strategy that we did not use here to improve ethnic diversity in trial participation is geotargeting of social media advertisements in geographic areas with an ethnically diverse population [22]. The multistage follow-up strategy and stepped approach to incentives (eg, an additional £12 if completed within 24 hours at 6 months) undertaken throughout the iDEAS trial were also informed by previous research [11]. The need to have ongoing strategies to detect participant deception in web-based studies and trials is also supported in other studies, and other strategies recommended beyond those we used are to check participant IDs during onboarding and undertake IP address checks [13].

### Limitations

This study offers valuable insights for researchers conducting web-based or remote RCTs, but it is not without limitations. The cost per participant is calculated for different sociodemographic groups to demonstrate the relative increase in costs required to recruit a balanced sample. However, this stepped increase in costs is conflated by narrowing the focus to smaller groups in the population. For example, we would expect that each participant from ethnic minority groups would cost more than each participant overall when simply dividing the cost by the number of participants, because there are proportionately fewer of them. Regardless, our estimates of comparative costs for different demographic groups across different recruitment methods may help other researchers who are planning future trials. Furthermore, this study does not consider costs related to setting up the trial, developing automation, designing materials for data collection and recruitment, and engaging with stakeholders to promote recruitment. These are additional upfront and ongoing costs that should be considered when costing RCTs. There are also 2 limitations related to the generalizability of these findings. Due to the very small numbers of some ethnic minorities, ethnicity was treated as White versus ethnic minority. Grouping all ethnic minority participants together in this way does not allow examination of different methods of recruitment for attracting different ethnic minorities. Furthermore, the Drink Less app is currently only available to those with an iOS device, and as such, iOS device ownership was an entry requirement for the trial. There are some sociodemographic differences in iPhone ownership: relative to Android devices, iPhone owners are younger, more likely to be women [23], and have higher average incomes [24].

### Conclusion

Most participants in this remote RCT were recruited through advertisements on social media (2483/5602, 44.32%), the NHS website (1961/5602, 35.01%), and through radio or newspapers (745/5602, 13.3%). Most recruitment methods oversampled participants who were more advantaged, women, and White. Targeted approaches through social media and radio advertising were successful in recruiting men but less successful in appealing to a more diverse demographic in terms of ethnicity and SES. There was evidence that the sequential approach taken to 6-month follow-up was successful, with 79.58% (4458/5602) follow-up rates at 6 months. This study offers recommendations for achieving balance in methodological challenges when conducting remote RCTs. Recruitment methods should be broad and targeted to achieve sociodemographic diversity. Automated emails with substantial financial incentives can achieve excellent follow-up rates of approximately 70%, but sequential offline follow-up can further boost retention by nearly 10% overall. SMS text messages can be a low-cost, low-effort way to improve follow-up rates. An important and broader takeaway is the importance of continuously monitoring, identifying, reacting to, and documenting new methodological challenges as they appear over the course of a trial. This is necessary not only to improve individual trials but also because pooling shared experiential learning can help research teams who are planning future trials.

## Abbreviations

ABC1: managerial, professional, and intermediate occupations
AUDIT: Alcohol Use Disorders Identification Test
C2DE: skilled, semiskilled, unskilled manual, and lowest-grade worked or unemployed
CAPTCHA: Completely Automated Public Turing test to tell Computers and Humans Apart
GP: general practitioner
iDEAS: iOS Drink Less, Evaluating the Effectiveness of an Alcohol Smartphone
NHS: National Health Service
PPI: public and patient involvement
RCT: randomized controlled trial
SES: socioeconomic status
UCL: University College London

## Data Availability

The data sets generated during and analyzed during this study are available on Open Science Framework [25].
